# Further evidence for *POMK* as candidate gene for WWS with meningoencephalocele

**DOI:** 10.1186/s13023-020-01454-0

**Published:** 2020-09-09

**Authors:** Luisa Paul, Katrin Rupprich, Adela Della Marina, Anja Stein, Magdeldin Elgizouli, Frank J. Kaiser, Bernd Schweiger, Angela Köninger, Antonella Iannaccone, Ute Hehr, Heike Kölbel, Andreas Roos, Ulrike Schara-Schmidt, Alma Kuechler

**Affiliations:** 1 Department of Pediatric Neurology, University Hospital Essen, University Duisburg-Essen, Essen, Germany; 2 Department of General Pediatrics, University Hospital Essen, University Duisburg-Essen, Essen, Germany; 3 Institute of Human Genetics, University Hospital Essen, University Duisburg-Essen, Essen, Germany; 4 Department of Radiology, University Hospital Essen, University Duisburg-Essen, Essen, Germany; 5 Department of Obestetrics and Gynaecology, University Hospital Essen, University Duisburg-Essen, Essen, Germany; 6 Center for Human Genetics, Regensburg, Germany / Department of Human Genetics, University of Regensburg, Regensburg, Germany; 7 Children’s Hospital of Eastern Ontario Research Institute, University of Ottawa, Ottawa, ON K1H 8L1 Canada

**Keywords:** *POMK*, Protein O-mannose kinase, Walker-Warburg syndrome, Alpha-dystroglycanopathy, Congenital muscular dystrophy, Meningoencephalocele

## Abstract

**Background:**

Walker-Warburg syndrome (WWS) is a rare form of alpha-dystroglycanopathy characterized by muscular dystrophy and severe malformations of the CNS and eyes. Bi-allelic pathogenic variants in *POMK* are the cause of a broad spectrum of alpha-dystroglycanopathies. *POMK* encodes protein-O-mannose kinase, which is required for proper glycosylation and function of the dystroglycan complex and is crucial for extracellular matrix composition.

**Results:**

Here, we report on male monozygotic twins with severe CNS malformations (hydrocephalus, cortical malformation, hypoplastic cerebellum, and most prominently occipital meningocele), eye malformations and highly elevated creatine kinase, indicating the clinical diagnosis of a congenital muscular dystrophy (alpha-dystroglycanopathy). Both twins were found to harbor a homozygous nonsense mutation c.640C>T, p.214* in *POMK*, confirming the clinical diagnosis and supporting the concept that *POMK* mutations can be causative of WWS.

**Conclusion:**

Our combined data suggest a more important role for *POMK* in the pathogenesis of meningoencephalocele. Only eight different pathogenic *POMK* variants have been published so far, detected in eight families; only five showed the severe WWS phenotype, suggesting that *POMK*-associated WWS is an extremely rare disease. We expand the phenotypic and mutational spectrum of *POMK*-associated WWS and provide evidence of the broad phenotypic variability of *POMK*-associated disease.

## Background

Encephalocele is a congenital malformation in which herniated meninges (with or without brain tissue) protrude outside the skull. The underlying cause has still not been completely elucidated [1]. Encephalocele is characteristic for some syndromal diseases, such as Knobloch syndrome (*COL18A1*), as well as certain ciliopathies such as Meckel-Gruber and Joubert syndromes, and several chromosomal aberrations [2]. Regarding diseases affecting proper glycosylation of alpha-dystroglycan, the manifestation of a meningoencephalocele has only rarely been associated with defects in known genes such as *POMT1* and *ISPD* [3, 4]. Geis and colleagues discussed the presence of an encephalocele as possibly an indicator of the presence of pathogenic *POMT1* mutations in terms of a phenotype-genotype correlation [3].

The *protein-O-mannosyl kinase* gene (*POMK,* OMIM *615247) encodes a protein involved in the glycosylation of the laminin-binding O-coupled carbohydrate chain of alpha-dystroglycan (α-DG). The α-DG in turn links the dystrophin complex via the sarcolemma to the extracellular matrix. *POMK* is expressed in various tissues including muscle, brain, retina, heart and kidney and is equally abundant in fetal and adult brain, heart and kidney tissues but reduced in skeletal muscle, with reduced expression at the end of the fetal period and an expression pattern predominantly in interstitial cells and blood vessels [5]. It is suspected that *POMK* plays an important role in the fetal development of myocytes, and indeed, embryonic *pomk* knockout zebrafish showed reduced embryonic motility and muscular dystrophy 3 days post fertilization [5]. In addition, *Pomk*-deficient zebrafish embryos showed small heads, delayed eye development, shortened and thickened tails and U-shaped somites as well as reduced embryo motility [5]. Notably, *Pomk-*deficient mouse models show severe, often lethal phenotypes with neuronal heterotopias in some brain areas, possibly as a consequence of defective neuronal migration [6]. The phenotypic presentation of both animal models accords with the hypothesis that POMK plays a significant role in muscle and nervous tissue, impacting on the differentiation of the respective cell types, and is in line with the concept of *POMK* as a gene occasionally associated with manifestation of brain malformations.

In humans, mutations within the *POMK* gene can lead to different alpha-dystroglycanopathy phenotypes ranging from the milder form (type MDDGC12 [7], OMIM #616094) to the most severe form, named Walker-Warburg syndrome (WWS, also named MDDGA12, OMIM #615249), which is a congenital muscular dystrophy associated with central nervous system and eye malformations. Variants in *POMT1* are the main cause of WWS. Like *POMK,* the *POMT1* gene also codes for an enzyme involved in the O-mannosylation pathway. The clinical picture of patients with *POMT1* variants includes neural tube defects ranging from meningocele to meningoencephalocele [8]. Compared to *POMT1*, *POMK* variants leading to WWS are rarer and either result in the expression of a shortened, incorrectly folded protein or interfere with catalytic function [9]. *POMK* genotype-phenotype correlations are complex because even mutations leading to expression of a (massively) shortened protein can result in a mild phenotype. However, the functional and physiological mechanisms explaining the phenotypic variability still remain unclear [5].

To date, only 14 patients with *POMK*-associated alpha-dystroglycanopathy due to 8 different mutations have been described [5, 7, 10–13] and two additional pathogenic or likely pathogenic variants are listed in the *POMK *database [https://databases.lovd.nl/shared/view/POMK; accessed 7 April 2020] . An encephalocele was reported for only one patient, a 19-week fetus (termination of pregnancy; TOP) by Jae and colleagues [11]. Here, we describe monozygotic twins each with occipital meningoencephalocele and homozygous nonsense mutation in *POMK.*

## Results

### Clinical details

**Fig. 1:**
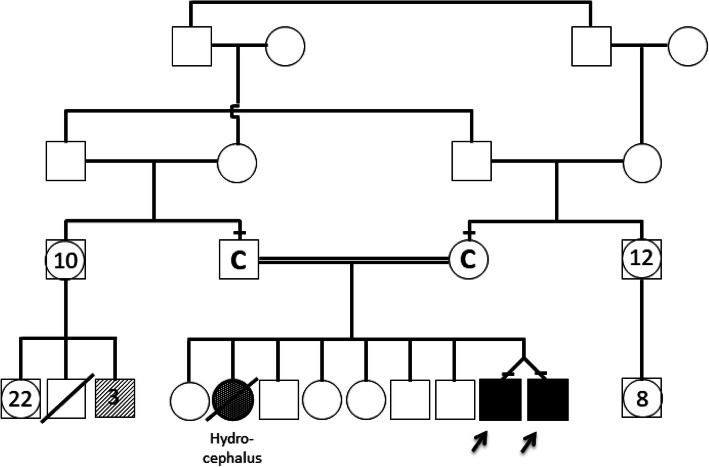
Pedigree. Healthy consanguineous parents. Six healthy siblings – one sibling died postnatally (congenital hydrocephalus). Three paternal first cousins (males) are affected by developmental delay (grey squares – no further information available)

The mother and father are healthy consanguineous parents (first cousins) having in total nine children: six are healthy and one died postnatally due to complications of hydrocephalus (no further data or material available). Further family history was unremarkable apart from three paternal nephews with unspecific developmental delay of unknown origin (Fig. 1).

**Fig. 2:**
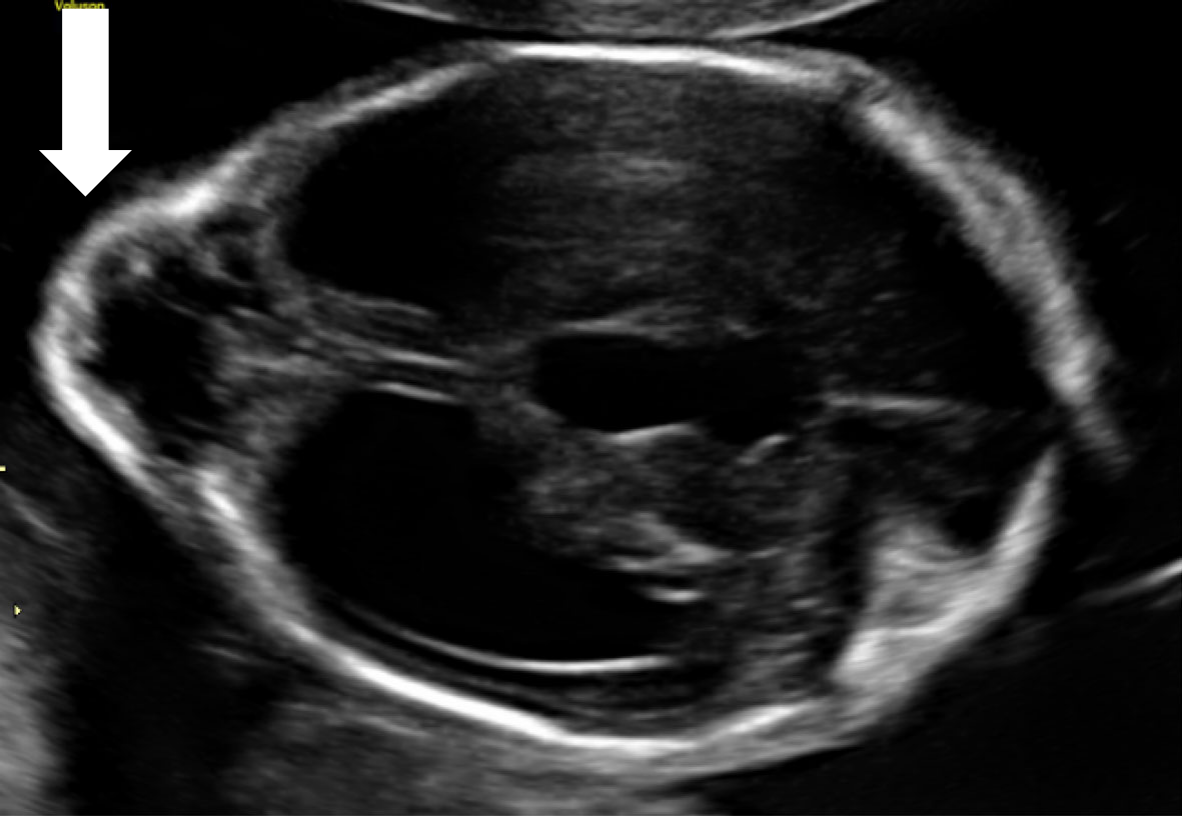
Prenatal ultrasound of the brain (22th week of pregnancy): occipital encephalocele (white arrow) and hydrocephalus

**Fig. 3:**
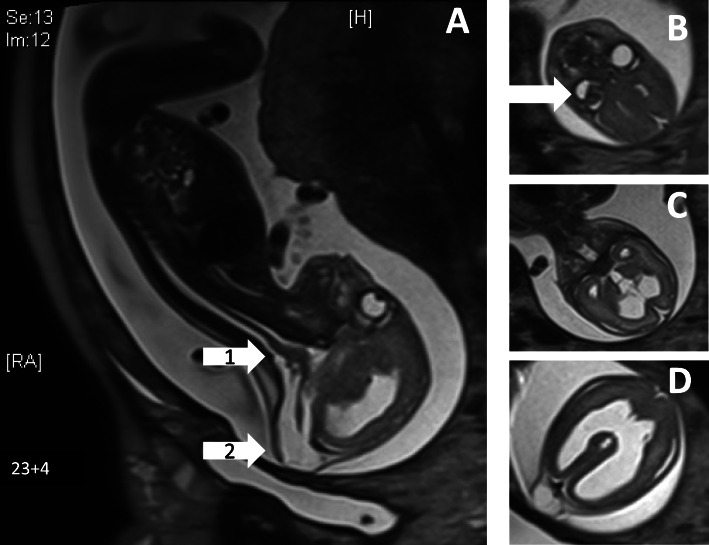
Prenatal MRI scan at 23 + 4 weeks of gestation; **a** hypoplastic cerebellum (white arrow 1), occipital meningocele (white arrow 2); **b** persistent hypoplastic primary vitreous body (white arrow); **c** + **d** internal hydrocephalus

**Fig. 4:**
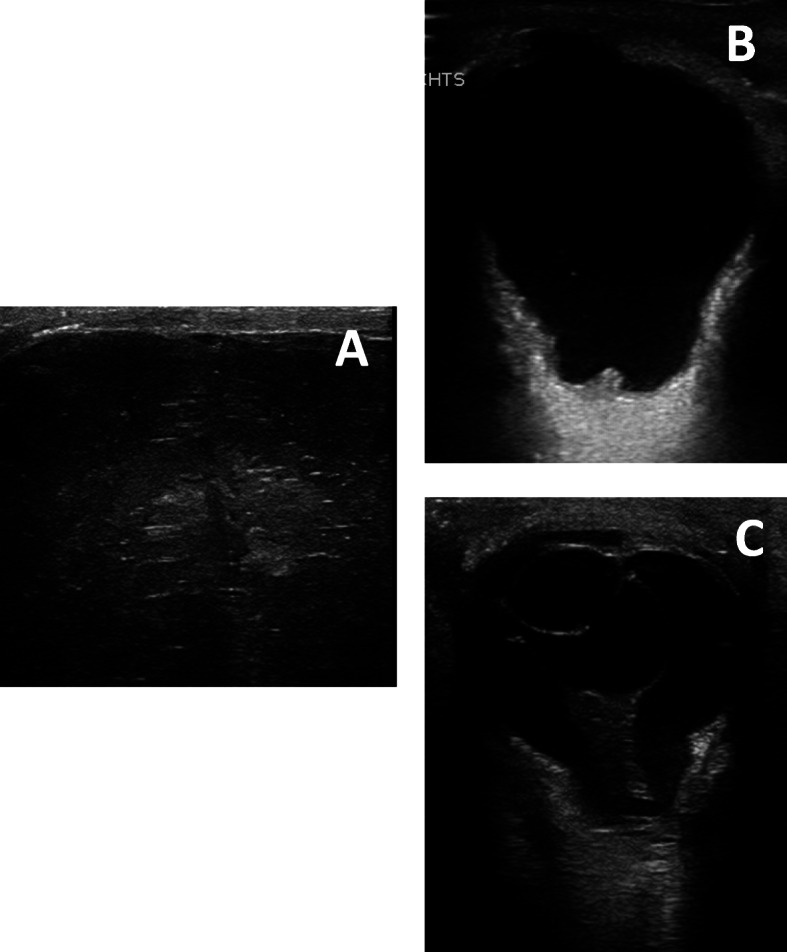
Postnatal ultrasound of the brain / eyes; **A** lissencephaly with polymicrogyria; **b** staphyloma; **c** persistent hypoplastic primary vitreous body

Prenatal ultrasonography screening in the 23rd week of pregnancy revealed the presence of occipital encephaloceles, hydrocephalus and cerebellar hypoplasia in both twins (Fig. 2). Prenatal MRI scans confirmed these findings (Fig. 3). Monochorionic-diamniotic male twins were born in gestational week 35 + 2 by planned caesarean section and because of progressive contractions. Birth parameters were in the normal range. Gemini 1 (G1): weight 2330 g (− 0.6 SD, 20th percentile), length 47 cm (30th percentile, − 0.3 SD), and occipitofrontal circumference 31 cm (8th percentile, − 1.3 SD). - Gemini 2 (G2): weight 2230 g (15th percentile, − 0.9 SD), length 47 cm (30th percentile, − 0.3 SD), and occipitofrontal circumference 30.5 cm (4th percentile, − 1.6 SD). Postnatally, additional eye malformations were observed by ultrasound and posterior ophthalmoscopy in both siblings and lissencephaly (Fig. 4). G1 showed a microphthalmos of the right eye and a coloboma dorsolateral of the left bulbus. G2 showed a bilateral persistent hyperplastic primary vitreous body and a posterior staphyloma of the left eye. The eye malformations led to blindness in both twins.

Brainstem evoked response audiometry (BERA) diagnosed severe sensorineural hearing loss in both patients.

**Fig. 5:**
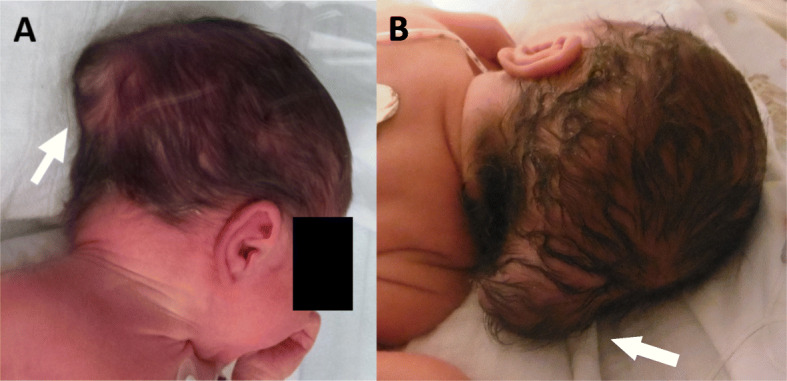
**a**-**b** postnatal examination of both twins revealed prominent occipital meningocele (white arrows)

Neuropediatric examination showed floppy infants with severe generalized muscle hypotonia, hyporeflexia, decreased spontaneous motor activity and muscular weakness observed when moving the muscles of the extremities against gravity. In addition to the ocular malformation, a prominent occipital protuberance was detected (histologically diagnosed as a meningoencephalocele) (Fig. 5).

Laboratory analysis showed a significant increase of the creatine kinase level to 7159 U/l (G1) and 8769 U/l (G2), respectively, with an accompanying elevation of transaminase levels and LDH.

**Fig. 6:**
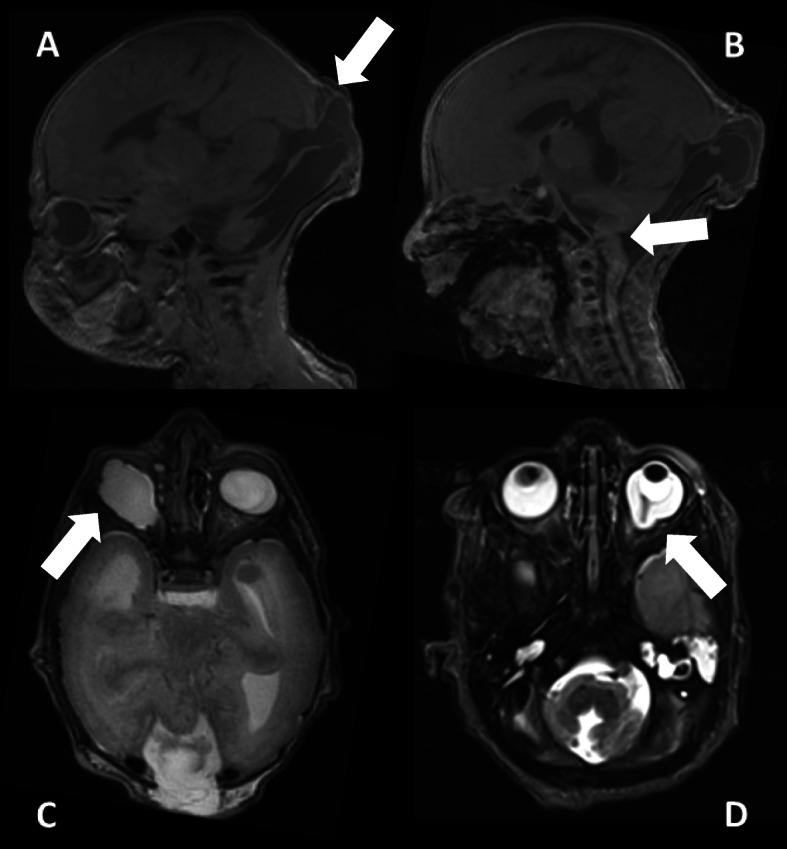
Postnatal cranial MRI scan (performed at 2nd / 3rd day); clinical findings: occipital meningocele with dorsal enlarged 4th ventricle (white arrow picture **a**), vermis hypo−/aplasia (white arrow picture **b**), generalized polymicrogyria-like cobblestone malformation, temporo-occipital subcortical band heterotopia, eye malformations (G1: microphthalmia with coloboma and caudal cyst, G2: persistent hypoplastic primary vitreous body and posterior staphyloma). **a**/**c** = G1, **b**/**d** = G2

MRI of the brain (Fig. 6) and spine, each performed on the 2nd/3rd day of life, showed a median occipital meningocele with dorsally opened fourth ventricle and hypo- to aplastic cerebellar vermis, a long narrowed thoracic myelon with dorsal attachment of single caudal fibers, internal hydrocephalus as well as dysgyria with generalized polymicrogyria-like cobblestone malformations and bi-temporo-occipital subcortical band heterotopia.

During the perinatal period, the meningoencephalocele was treated with neurosurgery and the hydrocephalus was treated with a ventriculo-peritoneal (VP) shunt. Both twins developed structural epilepsy with generalized seizures; G1 had repeated seizures with epileptic status refractory to antiepileptic therapy.

Both boys were provided with hearing aids. They received supportive therapy including physiotherapy and early remedial education and were cared for by our palliative care team. They had repeated hospitalizations due to therapy refractory epileptic seizures and severe respiratory infections. Psychomotor development was severely retarded and typical developmental milestones were not reached (even head control was not possible). Both boys could spontaneously move their heads, but the other extremities were tetraplegic. G2 was last measured at the age of 6 months [weight: 8430 g (0.44 SD), length 65.5 cm (− 0.95 SD)]. At the age of 17 months, G2 died of cardiorespiratory failure due to aspiration. Currently, at the age of 30 months, G1 continues to show no psychomotor development [weight: 16000 g (1.38 SD), length 94 cm (0.43 SD), occipitofrontal circumference: 44 cm (− 5.0 SD)].

### Molecular genetic analysis

In consideration of the clinical findings, a molecular panel analysis for genes related to WWS was performed including *FKRP, FKTN, ISPD, B3GNT1, COL4A1, LARGE, POMK* and *TMEM5*.

Due to the combination of severe CNS- and eye-malformations in combination with high CK-levels in these floppy infants, an alpha-dystroglycanopathy was considered. This assumption was supported by the presence of elevated serum CK levels. Panel sequencing resulted in the identification of a homozygous nonsense mutation [c.640C>T; p.Gln214*; NM_032237.4, OMIM: 615247] within the *POMK* gene in both twins. Sanger sequencing confirmed the homozygous *POMK* variant in both siblings and showed the parents to be heterozygous mutation carriers. Unfortunately, no material of the deceased sibling presenting with a hydrocephalus was available for mutational screening.

## Discussion

Walker-Warburg syndrome is a rare form of congenital muscular dystrophy (prevalence approximately 1:60,500) [14] with currently 18 known causative genes (*POMT1, POMT2, POMGNT1, FKTN, FKRP, LARGE1, ISPD, POMGNT2, GMPPB, DAG1, TMEM5, B3GALNT2, POMK, B3GNT1, DOLK, DPM1, DPM2, DPM3)* [15]. So far, only five families with WWS caused by variants in *POMK* have been described (in total eight individuals of whom four are terminated pregnancies; for details see families 3–7 in Table 1). All causative variants reported affected or diminished POMK activity, either by truncating the protein or impacting on the catalytic protein domain [5, 10–15].

**Table 1:** Clinical presentation and diagnostic characterization of individuals with pathogenic *POMK* mutations

	this study		Di Constanzo et al. [5],			Renesse et al. [10],		Jae et al. [11],		Preiksaitiene et al. [12],				Ardicli et al. [13],	Strang-Karlsson et al. [7],	
	family 1 patient 1^**#**^	family 1 patient 2^**#**^	family 2 patient 3	family 2 patient 4	family 3 patient 5^*^	family 4 patient 6^*^	family 4 patient 7^*^	family 5 patient 8^*^	family 5 patient 9^**#**^	family 6 patient 10^*^	family 6 patient 11^*^	family 6 patient 12^*^	family 7 patient 13^*^	family 8 patient 14	family 9 patient 15	family 9 patient 16
**c-DNA mutation**	c.640C>T	c.640C>T	c.325C>T	c.325C>T	c.286delT c.905T>A	c.325C>T	c.325C>T	c.410T>G c.773A>G	c.410T>G c.773A>G	c.136C>T	c.136C >T	c.136C>T	c.136C>T	c.401T>G	c.965C>T c.136C>T	c.965C>T c.136C>T
**protein mutation**	p.Gln214*	p.Gln214*	p.Gln109*	p.Gln109*	p.Phe96Phefs*19 p.Val302Asp	p.Gln109*	p.Gln109*	p.Leu13Arg p.258Arg	p.Leu13Arg p.258Arg	p.Arg46Ter	p.Arg46Ter	p.Arg46Ter	p.Arg46Ter	p.V134G	p.Pro322Leu p.Arg46Ter	p.Pro322Leu p.Arg46Ter
**age of onset**	neonatal	neonatal	infancy	infancy	neonatal	infancy	neonatal	neonatal	neonatal	neonatal	neonatal	neonatal	neonatal	childhood	childhood	childhood
**first signs**	in utero*:* cerebral malformation, opened dorsal 4th ventricle, encephalocele	in utero: cerebral malformation, agyri, encephalocele	floppiness and delayed walking at 18 months	floppiness and delayed walking at 18 months	in utero: macrocephaly, hydrocephalus	feeding problems, motoric development delayed	proximal weakness, little antigravity movements, hyporeflexia	*typical WWS*	in utero*:* ventriculomegaly/ hydrocephalus, absence of the falx cerebri and cerebellar tentorium, occipital encephalocele	in utero*:* ventriculo-megaly, thin cortex, hydro-cephalus, macro-cephaly	in utero*:* ventriculo-megaly	in utero*:* ventriculo-megaly (lateral / 4th ventricle)	in utero*:* hydrocephalus, ventriculo-megaly, suspected aplasia of cerebellar vermis	muscle weakness, easy fatigue, clumsiness, difficulty running and climbing	hip / neck cramps, growing pain	thigh stiffness, cramps thigh/neck
**muscle weakness (locali-sation)**	CMD, postnatal reduced spontaneous motor movement, lifting limbs against gravity	CMD, postnatal reduced spontaneous motor movement, lifting arms against gravity	proximal weakness, calf pseudo-hypertrophy, mild facial weakness	posture and gait affected	CMD, spontaneous motility absent at 7 months	CMD, proximal weakness (difficulties climbing stairs and running)	proximal weakness, never able to sit, roll on the side with 2 years	*typical WWS*	n/a	n/a	n/a	n/a	n/a	CMD, muscle weakness age of 12), calf hypertrophy, proximal muscle weakness, Gowers sign	CMD, proximal weakness, calf hyper-trophy	CMD, proximal weakness
**CK level**	7159 U/l	8769 U/l	1090 U/l	1420 U/l	3985 U/l	1238 U/l	1810 U/l	*typical WWS*	n/a	n/a	n/a	n/a	n/a	2400 U/l	1000-4000 U/l	6800 U/l
**biopsy findings**	n/a	n/a	n/a	dystrophic, cell death and regeneration positive for all markers tested dystrophin, utrophin, merosin, dysferlin, sarco-glycans and b-dystro-glycan	muscle fibers are absent	myopathic pattern with normal dystrophin expression (9 months) re-biopsy (age of 4): laminin, alpha 2, merosin reduced	myopathic pattern and merosin deficiency	n/a	n/a	n/a	n/a	n/a	n/a	mild dystrophic changes, increase in nuclei, degenerating and regenerating fibers, focal endomysal fibrosis immunofluorescent analysis: laminin alpha 2 pos., alpha-dystroglycan neg., dystrophin/ sarcoglycan pos.	normal	moderate chronic myopathic changes, small groups of regenerating fibers, sparse inflammatory cell infiltrates, alpha-dystroglycan deficiency, normal merosin immunolabelling
**MRI findings**	meningocele, opened 4th ventricle, hypoplastic vermis cerebellum, small myelon, arachnoid cyst	meningocele, opened 4th ventricle, hypoplastic vermis, lissencephaly, small myelon, arachnoid cyst	cysterna magna	temporal lobe arachnoid cyst	cobblestone lissencephaly, agenesis of the corpus, severe cerebellar vermis hypoplasia	symmetric cerebral white matter changes (15 months)	hypomyelination	aquaeductal stenosis, hydrocephalus, agyria, cerebellar and brainstem hypoplasia, Arnold-Chiari malformation	n/a	n/a	n/a	n/a	n/a	cerebellar hypoplasia, cortical disorganization, brainstem hypoplasia, cerebellar cortical microcysts, bilateral hippocampal incomplete rotation	n/a	n/a
**ocular findings**	anophthalmus (right eye), blindness, cataract (left), staphyloma (left)	congenital cataract, bilateral hypoplastic corpus vitreum, lagophthalmos bilateral	none	none	glaucoma (right eye), bilateral retinal degeneration	eyes appeared large (corneal diameter 11,5 mm) – no criteria for megalo-cornea	reduced visual acuity	micro-ophthalmia, persistent hyperplasic primary vitreous body, myopia, cataract	cataract, coloboma	none	none	none	none	none	none	none
**additional information**	pathological EEG with delta-waves, tonic-clonic seizures, bilateral sensorineural hearing loss	pathological EEG with multifocal pathological EEG-potentials, seizures, bilateral sensorineural hearing loss, patent foramen ovale	hyporeflexia		hypotonia, bilateral sensorineural hearing loss,delayed psycho-motor development, tonic seizures	sensorineural hearing loss	BERA: moderate hearing impairment, contractures knees/hips							mirror movements (hands) since infancy	birth asphyxia due to placental abruption, high frequency hearing loss	preterm birth (placenta previa) Weakened function of left ventricle (age 12)
**latest check up**	30 months: severe motor and verbal developmental disorder: hypersalivation, no movement of the head, no active movement of the extremities or active language	17 months: cardiopulmonary resuscitation with exitus letalis due to an aspiration	still ambulatory at 25	at the age of 13 years: climbs stairs without support	death at the age of 4 years	at the age of 17 years: wheelchair, at the age of 21 years: lost ambulation, not able to stand, eat or drink without support	at the age of 10 years: scoliosis, pathologic pulmonary function (VC:36%)	death at the age of 3 years	TOP (19 weeks of gestation)	died during labor (32 weeks of gestation, TOP)	TOP (16 weeks of gestation)	TOP (14/15 weeks of gestation)	TOP (19 weeks and 6 days of gestation) autopsy: massive hydrocephalus, aplasia of the cerebellar vermis	at the age of 19 years: mild learning difficulties, reduced deep tendon reflexes, pes cavus deformity	calf hypertrophy, mild lumbar lordosis, slightly winged scapulae, brisk tendon reflexes in upper extremities	calf hypertrophy, mild lumbar lordosis, slightly winged scapulae, brisk tendon reflexes in upper extremities, problems walking on heels

**C**olumns of individuals with severe WWS phenotype are marked *; #: WWS+ encephalocele

The clinical manifestation of patients with pathogenic variants in *POMK* is rather broad (see introduction) and a varying spectrum of clinical manifestations and severity can even be observed within the subgroup of *POMK*-related WWS (classified as such by the authors/clinicians).

Renesse et al. described two siblings of a consanguineous family with a homozygous *POMK* nonsense mutation (c.325C>T, p.Q109X). Both siblings had secondary microcephaly, muscular hypotonia, feeding problems and developmental delay. In addition, they presented with hypomyelinization of the brain, mild hearing loss and intellectual disability [10]. The 15-year-old sibling has developed joint contractures, neuromuscular scoliosis and nocturnal hypoventilation. The 22-year-old sibling has used a wheelchair since the age of 17 and is dependent on comprehensive help in her everyday life. Ocular abnormalities were found in the form of large bulbi with reduced visual acuity, but did not meet the criteria of a megalocornea in this patient [10]. Given that the clinical presentation of these *POMK* siblings manifests in the spectrum between the milder MDDGC12 and the most severe MDDGA12 phenotype, these cases might be classified as MDDGB12.

Di Costanzo et al. reported on three patients from two different families with pathogenic *POMK* mutations. Patients from one family presented with the same homozygous *POMK* nonsense mutation (c.325C>T, p.Q109X) as the patients described by Von Renesse et al. [5, 10]. However, clinically, the patients described by Di Costanzo et al. had a milder limb-girdle muscular dystrophy phenotype in line with MDDGC12: both patients showed their first symptoms in infancy but are still able to walk at the age of 25 and 13 years, respectively. They show no ocular malformations and their IQ is slightly below average (IQ 80 and 83) [5]. In contrast, the second family characterized by Di Costanzo et al. had a compound heterozygous mutation (combination of frame shift and missense mutation, c.286delT, p.Phe96Phefs*19 and c.905T>A, p.Val302Asp) and presented clinically with WWS, similar to our two cases. The affected child was diagnosed in utero with macrocephaly and hydrocephalus. Postnatally, muscular hypotonia was present, as well as other CNS malformations including cobblestone lissencephaly, corpus callosum agenesis, vermis aplasia further complicated by eye malformations (glaucoma, retinal degeneration) and severe sensorineural hearing loss. The child showed a severe psychomotor developmental disorder, developed tonic seizures and died at the age of 4 years [5]. Jae et al. described additional compound heterozygous missense mutations in the *POMK* gene (p.Leu137Arg, p.Gln258Arg) in a patient with typical symptoms of WWS [11]. The second child of the family had severe brain malformations as well as an occipital encephalocele and died prenatally [11].

Very recently, Preiksaitiene et al. reported on two families with in total four WWS patients of which three were TOPs: brain malformations with hydrocephalus and ventriculomegaly due to a *POMK* nonsense mutation were detected in utero [12]. One mildly affected *POMK* patient (caused by a homozygous missense mutation) presenting with mirror movements of the hands was described by Ardicli et al.: the initial symptoms manifested during childhood with muscle weakness, easy fatigability, clumsiness and difficulties running and climbing. At the age of 12, proximal muscle weakness with calf hypertrophy was detected. On the MRI scan, several brain malformations were identified – surprisingly only associated with mild learning difficulties [13]. In addition, Strang-Karlsson et al. reported on a family with two siblings with a homozygous *POMK* missense mutation resulting in mild congenital muscular dystrophy: during childhood hip and neck cramps (triggered by yawning) were described together with proximal muscle weakness with calf hypertrophy. Investigation of a muscle biopsy obtained from one patient revealed normal histological findings whereas the biopsy from the sibling showed moderate chronic myopathic changes with small groups of regenerating fibers and sparse inflammatory cell infiltrates [7].

Based on our findings as well as previous findings of meningoencephaloceles in patients with *POMT1* and *ISPD* mutations, we recommend an initial laboratory analysis of CK in newborns which present clinically with the combined symptoms of muscular weakness and meningoencephalocele [3, 4].

POMK is an atypical kinase that phosphorylates the 6-position of O-mannose after mannose has been modified by both GTDC2 and B3GALNT2 (two proteins encoded by genes leading to overlapping neurological phenotypes). The glycan structure resulting from POMK-modulated phosphorylation appears to be relevant for binding to the extracellular matrix (ECM) [10, 16]. Although the basic biochemical function of POMK is well understood, further research on larger *POMK* patient populations is needed to improve understanding of the phenotypic variability, which might be caused by the activation of compensatory mechanisms (warranting proper protein glycosylation and ECM assembly) and/ or the presence of further molecular genetic alterations of relevance as modifiers.

## Conclusion

Given that encephaloceles are occasionally associated with other genetic defects causative of alpha-dystroglycanopathies, including genes encoding for proteins involved in O-mannosylation of α-DG such as *POMT1* [3], the presence of a meningoencephalocele in our *POMK* patients supports the concept that perturbed post-translational modification of α-DG has a detrimental impact on α-DG-function and affects correct maturation of the neural tube during fetal development. Our combined clinical and genetic findings thus expand the clinical spectrum of *POMK* patients and classify *POMK* as candidate gene for meningoencephalocele.

## Methods

### Study aim, design and setting of the study

The study aimed to combine clinical and diagnostical findings obtained in different patients with *POMK* mutations. The study took place at the university hospital Essen.

### Characteristics of participitans

We analysed two monozygous twins with a mutation in *POMK*. We compared different patients published before and focused on mutations in c-DNA and protein level, age of onset, first symptoms, muscle weakness (localisation), CK level, biopsy findings, MRI findings, ocular findings, additional information and latest check-up.

## Data Availability

All data generated or analysed during this study are included in this published article.

## Abbreviations

a-DG: alpha-dystroglycan
BERA: Brainstem evoked response audiometry
B3GALNT2: Beta-1,3-N-Acetylgalactosaminyltransferase 2
B3GNT1: N-acetyllactosaminide beta-1,3-N-acetylglucosaminyltransferase
CDG: Congenital disorder of glycosylation
CK: Creatine kinase
CMD: Congenital muscle dystrophy
CNS: Central nervous system
COL18A1: Collagen type XVIII alpha 1 chain
COL4A1: Collagen type IV alpha 1 chain
DAG1: Dystroglycan 1
DOLK: Dolichol kinase
DPM1: Dolichyl-Phosphate Beta-D-Mannosyltransferase Subunit 1
DPM2: Dolichyl-Phosphate Beta-D-Mannosyltransferase Subunit 2
DPM3: Dolichyl-Phosphate Beta-D-Mannosyltransferase Subunit 3
ECM: Extraceullar matrix
FKRP: Fukutin-related protein
FKTN: Fukutin
GMPPB: GDP-Mannose Pyrophosphorylase B
GTDC2: Glycosyltransferase-like domain containing 2
ISPD: Isoprenoid synthase domain-containing protein
LARGE: Like-glycosyltransferase
MDDGA12: Muscular dystrophy-dystroglycanopathy Type A 12
MDDGC12: Muscular dystrophy-dystroglycanopathy Type C 12
OMIM: Online mendelian inheritance in man
POMGNT1: Protein O-linked mannose N-acetylglucosaminyltransferase 1
POMGNT2: Protein O-linked mannose N-acetylglucosaminyltransferase 2
POMK: Protein-O-Mannose Kinase
POMT1: Protein-O-mannosyltransferase 1
POMT2: Protein-O-mannosyltransferase 2
SHH: Sonig hedgehog gene
TMEM5: Transmembrane protein 5
TOP: Termination of pregnancy
VC: Vital capacity
VP: Ventriculo-peritoneal shunt
WWS: Walker-Warburg syndrome
